# Musical tasks targeting preserved and impaired functions in two dementias

**DOI:** 10.1111/nyas.12616

**Published:** 2015-03-13

**Authors:** Andrea R Halpern, Hannah L Golden, Nadia Magdalinou, Pirada Witoonpanich, Jason D Warren

**Affiliations:** 1 Psychology Department, Bucknell UniversityLewisburg, Pennsylvania; 2 Dementia Research Centre, UCL Institute of Neurology, University College LondonLondon, United Kingdom; 3 Department of Molecular Neuroscience, UCL Institute of Neurology, University College LondonLondon, United Kingdom

**Keywords:** Alzheimer's disease, Parkinson's disease, auditory imagery, executive function

## Abstract

Studies of musical abilities in dementia have for the most part been rather general assessments of abilities, for instance, assessing retention of music learned premorbidly. Here, we studied patients with dementias with contrasting cognitive profiles to explore specific aspects of music cognition under challenge. Patients suffered from Alzheimer's disease (AD), in which a primary impairment is in forming new declarative memories, or Lewy body disease (PD/LBD), a type of parkinsonism in which executive impairments are prominent. In the AD patients, we examined musical imagery. Behavioral and neural evidence confirms involvement of perceptual networks in imagery, and these are relatively spared in early stages of the illness. Thus, we expected patients to have relatively intact imagery in a mental pitch comparison task. For the LBD patients, we tested whether executive dysfunction would extend to music. We probed inhibitory skills by asking for a speeded pitch or timbre judgment when the irrelevant dimension was held constant or also changed. Preliminary results show that AD patients score similarly to controls in the imagery tasks, but PD/LBD patients are impaired relative to controls in suppressing some irrelevant musical dimensions, particularly when the required judgment varies from trial to trial.

## Introduction

Music making is thought to be universal in human cultures. Music does not communicate messages in the same sense as does language, but it may have provided our ancestors, and us today, outlets for expressing emotion, play, worship of the divine, and other important aspects of social interaction and personal well-being.^1^ Musical ability is not uniformly distributed among people; for instance, the ability to match pitch accurately with one's voice is deficient in 10–20% of the population.^2^ However, complete inability to appreciate basics of music such as pitch relationships—amusia—is quite rare as a congenital condition (3–4% of the population^3^). Clearly, many people like music, and participation in music into later adulthood is quite common, both informally and in formal groups such as choirs. One recent study by Chorus America estimated that approximately 270,000 choirs operate in the United States, including professional, civic, school, and religious groups, involving over 42 million Americans, of whom approximately 32 million are adults.^4^

This attraction to music has prompted research-ers and music therapists to study how well musical abilities might persist in the face of challenge from neurodegenerative disorders. The belief that music perception and appreciation might be relatively spared in at least some neurological disorders is widespread. The literature has some remarkable case studies,^5^,^6^ and organizations such as Music & Memory advocate for bringing music to the lives of people with cognitive disorders. Cognitive scientists are also interested in using data from people with impairments to shed light on the cognitive and perceptual systems that support arts processing in healthy individuals. For instance, examining people with cognitive disorders such as Alzheimer's disease (AD) allows us to understand how much art or music appreciation depends on memory, language, or visuospatial skills^7^ and the relationship between cognitive impairments and decoding the emotional message in music.^8^ These understandings can help shape therapies for people with these disorders. As an example, to the extent that AD sufferers can recognize music from their past and even perform it, this contrasts with the compromised ability to learn and remember new music.^9^ Thus, therapists might want to tailor programs to emphasize music from the patient's premorbid life.

In this study, we sought to examine two dementias that we thought would have contrasting profiles of musical impairment. “Dementia” is used very generally to designate any syndrome of progressive cognitive deterioration: different dementia diseases have distinct etiologies and characteristic neuroanatomical profiles, with implications for both basic research questions and therapies alluded to above. We present preliminary results of studies in two canonical and distinctive dementia syndromes: AD and Lewy body dementia (LBD), which lies on a clinical and pathological continuum with Parkinson's disease.

We chose these two dementias because although both are common neurodegenerative disorders of later life, they contrast with one another in several important respects. AD in most cases initially affects the hippocampus, its posterior projection zones, and adjacent temporal lobe cortex, and is associated with compromise of acetylcholine transmission systems. Impairment in forming new memories is the foremost symptom of AD, and other memory functions are compromised as well.^10^–^13^ But (importantly for the current purposes) early perceptual processing areas are relatively less affected in AD. Lewy body pathology often affects the subcortical basal ganglia initially, with primary impact on dopamine transmission pathways, producing a syndrome of Parkinson's disease. Basal ganglia projection zones in frontal and other cortical regions become affected, and cognitive impairment commonly supervenes in Parkinson's disease (Parkinson's disease dementia, PDD^14^); however, cognitive decline may be the leading feature of the pathological process in the syndrome usually designated as LBD.^14^,^15^ The cognitive phenotype of PDD/LBD is variable^15^–^17^ but is frequently characterized by early prominent visual perceptual alterations (in particular, hallucinations) and attentional and executive dysregulation, although (in contrast to AD) episodic memory may be relatively spared. Patients with LBD typically have trouble organizing tasks, keeping track of recency and frequency, and dividing attention.

Many musical abilities have been studied in AD, as noted,^9^ but one aspect of musical memory has not so far been examined: auditory imagery. Most people report they can “hear” tunes in their head and are fairly accurate in reproducing the tempo, relative pitch, and even absolute pitch of tunes they know^18^ and can also imagine more complex qualities such as timbre.^19^ Imagery feels like an interaction between memory and perception, and indeed, a network comprising frontal memory areas and secondary auditory perception areas is active when people are imagining music (and is more active, the more vivid the experience).^20^ Because the perceptual processing areas are relatively preserved in AD, we gave patients and controls a mental pitch comparison task (as well as an actual pitch comparison task). In contrast to their documented memory problems in other areas, we predicted that this type of supported memory would be relatively preserved in AD patients.

Executive function in PD/LBD has typically been examined in verbal and visual domains.^17^ In extending the profile of executive dysfunction to the auditory/musical domain, we asked whether patients could suppress an irrelevant musical dimension. We chose pitch and timbre (musical instruments) as the dimensions of interest. These two musical qualities interact perceptually in that perception of one influences the perception of the other. As Krumhansl and Iverson^21^ showed, if you ask people to quickly say whether two notes are the same pitch, people are slower to say “yes” to identical notes if the timbre of the pitch changes, compared to no timbre change (this task is also known as a Garner task after the originator, Wendell Garner). The same is true when subjects compare two timbres. Because pitch and timbre interact, it requires active suppression to ignore the irrelevant dimension. Suppression is an executive function, so we therefore hypothesized that PD/LBD patients would be more prone to interference from one dimension to the other compared to controls, even in the relatively simple Garner task. We thought this would be particularly evident when type of trial (judging of pitch or timbre) was randomized rather than blocked. This task requires set shifting in addition to suppression, thereby requiring the use of not one but two executive functions.

## Methods

### Participants

For the imagery study, eight consecutive patients (three female) fulfilling clinical criteria for AD with predominant episodic memory loss and additional cognitive dysfunction^10^ were recruited and passed screening for the imagery task along with 18 healthy age-matched controls (10 female) with no psychiatric or neurological history. Ten consecutive patients (three female) meeting current consensus criteria for PDD/DLB^13^,^15^ were recruited and passed screening for the musical dimensions task along with 12 (seven female) aged-matched healthy control participants with no psychiatric or neurological history. Musical training varied (ranging from no experience to professional musician) but was matched between patient and control groups in terms of years of training. All participants gave informed consent in accordance with the declaration of Helsinki and local ethics approval committee. All PDD/DLB patients were receiving dopaminergic therapy, which induced definite but moderate fluctuation, and most of both the PDD/DLB and AD patients were receiving anticholinergic treatment for cognitive decline at the time of testing. All of the PDD/DLB and five of the AD patients (three did not participate because of time constraints) underwent a general neuropsychological assessment (Table1).

**Table 1:** General demographic, clinical, and neuropsychological data for patient groups

Characteristics	AD	PDD/DLB
General demographic		
No. (male:female)	5:3	7:3
Age (years)	72.0 (7.3)	72.4 (7.0)
Musical training (years)	5.8 (3.6)	3.7 (4.3)
Neuropsychological assessment		
WASI verbal IQ	108.2 (10.4)	104.6 (9.9)
WASI performance IQ	99.6 (19.3)	85.6 (11.2)
NART predicted IQ	118.6 (7.1)	109.1 (11.4)
Episodic memory		
RMT faces	−1.3 (1.0)	−0.5 (1.7)
RMT words	−2.5 (1.2)	−1.3 (1.4)
Executive skills		
WMS-R digit span forward	0.3 (0.9)	0.1 (1.0)
WMS-R digit span reverse	0.1 (0.7)	−0.4 (1.0)
D-KEFs Stroop color	−1.4 (1.7)	−1.5 (1.3)
D-KEFs Stroop word	−0.9 (2.0)	−0.9 (1.7)
D-KEFs Stroop interference	−1.1 (1.5)	−1.5 (1.3)
Verbal skills		
GNT	0.5 (1.3)	0.7 (1.4)
Posterior cortical skills		
GDA^a^	−0.4 (1.1)	−1.3 (0.7)
VOSP object decision	−1.4 (1.1)	−1.7 (0.6)

a One of the PDD/DLB patients could not complete this subtest.

Note: Mean (standard deviation) shown for demographic characteristics and *Z*-scores are shown for neuropsychological tests unless otherwise stated.

Abbreviations: AD, patients with a diagnosis of Alzheimer's disease; D-KEFS, Delis Kaplan Executive System; DLB, patients with a diagnosis of Dementia with Lewy bodies; GDA, Graded Difficulty Arithmetic; GNT, Graded Naming Test; NART, National Adult Reading Test; PDD, patients with a diagnosis of Parkinson's disease dementia; RMT, Recognition Memory Test; VOSP, Visual Object and Spatial Perception Battery; WASI, Wechsler Abbreviated Scale of Intelligence; WMS-R, Wechsler Memory Scale Revised.

### General screening task

All participants were screened for the ability to distinguish between two pitches. Participants were required to make a same/different judgment for note pairs (1-s duration and interstimulus interval) varying from one to five semitones, with 10 same and 10 different trials, with 80% accuracy. Six controls, five PDD, and one AD patient were excluded on this basis. A further screening test for the imagery task was administered to see if participants could correctly identify whether the second note was higher or lower than the first for two presented notes: half were higher and half lower. Pitch differences again varied from one to five semitones. The 80% criterion was also applied to this task; however, none of the participants who passed the first same/different screening task then went on to fail the higher/lower task.

### Musical dimensions task

Stimuli consisted of three possible pitches (F, G, A) and six different sampled instruments (clarinet, flute, French horn, oboe, tenor sax, trumpet, and violin), to implement the timbre dimension. Sound pairs were presented with a duration of 1 s with 1-s gaps between notes. Stimuli were presented via Cogent v1.32 extension for Matlab 2012a using a notebook computer and closed-ear headphones. Sounds were presented at an easily audible level for all participants (>70 dB). For all blocks, participants were instructed to listen to the sounds and watch the screen. After sounds were presented, a question screen with a reminder of which element to judge (“Was the INSTRUMENT/PITCH of the notes the same (SAME) or different (DIFF)?”) appeared, and participants were instructed to press a button for same or different (labeled “SAME” or “DIFF”). Where timbre pairs were different, instruments were matched for note register. Reaction times were measured from the onset of the question screen, and responses were recorded for offline analysis. Different sound pairs were presented dependent on condition, as detailed below:

Block 1: pitch-only judgments. Sound pairs were presented with instrument fixed within pairs, with six items of the same pitch and six items of different pitches.

Block 2: timbre-only judgments. Sound pairs were presented with pitch fixed between pairs, with six same and six different items.

**Figure 1:**
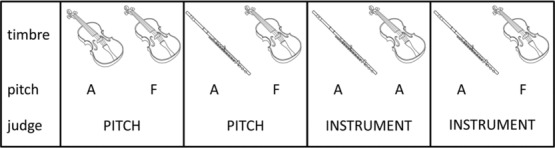
Schematic of four conditions presented in Block 3, reflecting the possible change of two elements: (1) type of judgment and (2) whether the irrelevant dimension was fixed or changed. This resulted in four subtypes of trials: pitch judgment with instrument fixed within pairs; pitch judgment with instrument change within pairs; instrument judgment with pitch fixed within pairs; and instrument judgment with pitch change within pairs.

Block 3: blocked presentation, altering irrelevant dimension. There were four conditions presented in this block, reflecting the possible change of two elements: (1) type of judgment and (2) whether the irrelevant dimension was fixed or changed. This resulted in four subtypes of trials: pitch judgment with instrument fixed within pairs; pitch judgment with instrument change within pairs; instrument judgment with pitch fixed within pairs; instrument judgment with pitch change within pairs. Conditions are schematized in Figure1. Type of judgment was blocked, with a reminder screen showing which judgment to make (INSTRUMENT or PITCH) during sound presentation. Participants started with instrument judgments, then pitch. Between the two blocks, participants were instructed that the judgment would change and to pay attention to the reminder screen so as to make the correct judgment. Six same and six different pairs were presented for each condition, giving a total of 48 trials. Within each judgment block, trials were pseudorandomized (each participant was presented with the same prerandomized order) so that whether the irrelevant dimension was fixed or changed was unpredictable.

Block 4: mixed presentation, altering irrelevant dimension. The same conditions as Block 3 were used, but type of judgment was also randomized across all trials; therefore, the participants were required to pay attention to the reminder screen for each trial to know which judgment to make.

### Tune imagery screening task

Because the task used well-known tunes that participants needed to retrieve from memory, each participant was screened for familiarity of tunes. We presented 78 tunes of which half were famous tunes thought to be familiar to the participant cohort and half were in reversed order (and thus unfamiliar). Participants had to judge whether each tune was familiar or unfamiliar; for each correct “familiar” judgment, a three-alternative forced-choice task of tune–title matching was presented. If participants could not correctly identify 20 tunes, they were excluded from further participation; however, none of either experimental group reached exclusion criteria. Twenty of the known tunes were selected to create a personal playlist for each participant in which only tunes that were familiar to the individual were presented.

### Main task

In the perception condition, each participant was presented with the initial phrases of 20 familiar tunes from his or her personal playlist; in approximately half, the second note was higher than the first and vice versa. Tunes were presented via headphones through a notebook laptop using the Cogent v1.32 extension for Matlab 2012a. Presented on screen were the title of the song and the lyrics of the excerpts. The first two notes were highlighted in blue (if there were two notes in the first word, just the first word was highlighted). Participants were instructed to respond with a button press to indicate whether the second note was higher or lower in pitch than the first. The whole tune excerpt was played, but participants were told to respond as soon as they knew the answer and not to worry about interrupting the tune.

In the imagery condition, the same tunes as in the perception condition were presented, this time with the title and the lyrics on screen but no auditory input. Participants were instructed to think of the tune in their head, without singing or humming, and indicate whether the second note was higher or lower than the first via the same button press. Again they were told to respond as soon as they knew the answer. Response and reaction times were stored for offline analysis.

## Results

### Musical dimensions

Patients did not significantly differ from controls in terms of gender distribution (*χ*^2^ = 1.8, *P* = 0.18), age (*t* = −0.83, *P* = 0.42), or years of musical training (*t* = 0.17, *P* = 0.87). In regard to accuracy, years of musical training significantly predicted performance (*β* = 4.3, CI = 3.5–11.0); however, age did not (*β* = 0.03, CI = −0.1 to 0.001). Because of multiple levels in the conditions, Wald tests of coefficients were used to indicate a significant main effect of condition (*F*(8,30) = 31.0, *P* < 0.001) but not patient group (*F*(1,30) = 0.41, *P* = 0.53). There was also no significant interaction between patient group and condition (*F*(8,30) = 0.96, *P* = 0.49).

**Figure 2:**
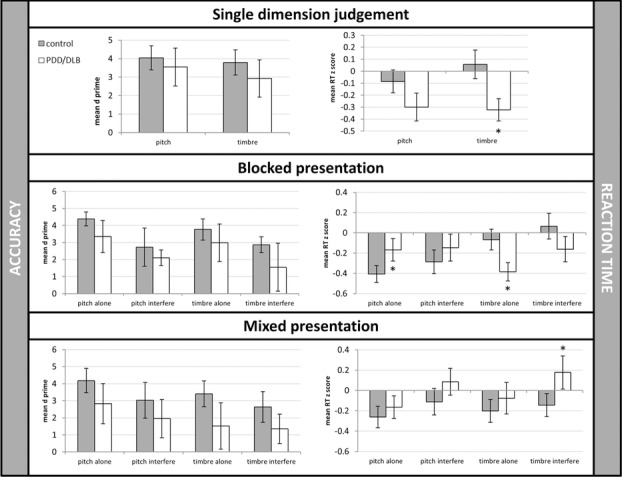
Accuracy and reaction times in PDD/DLB patients and controls. *P < 0.05.

We next examined response times, our measure of primary interest. Because PD and age can affect speed of responses, all reaction times were converted to *Z*-scores so that response times were normalized. A regression model considered age, years of training, condition, and patient status. This analysis revealed a significant influence of age (*β* = 0.005, CI = −0.008 to −0.002, *P* = 0.003) but not years of musical training (*β* = 0.001, CI = −0.004 to 0.002, *P* = 0.513). Wald tests revealed no main effect of patient group (*F*(1,21) = 1.77, *P* = 0.20) but a significant main effect of condition (*F*(8,21) = 58.22, *P* < 0.00001). There was a significant interaction between condition and patient group for RT performance (*F*(8,21) = 7.00, *P* = 0.0002). Further Wald tests to determine which differences seemed to be driving the interaction showed that patients and controls differed significantly in their reaction times for single-dimension–only timbre discrimination (block 2: *t* = −2.5, *P* = 0.02), blocked presentation pitch with no irrelevant dimension (block 3a: *t* = 2.17, *P* = 0.04), timbre with no irrelevant dimension (block 3c: *t* = −3.58, *P* = 0.002), and mixed-presentation timbre with irrelevant dimension (block 4d: *t* = 2.33, *P* = 0.03; Fig.2).

### Imagery task

**Figure 3:**
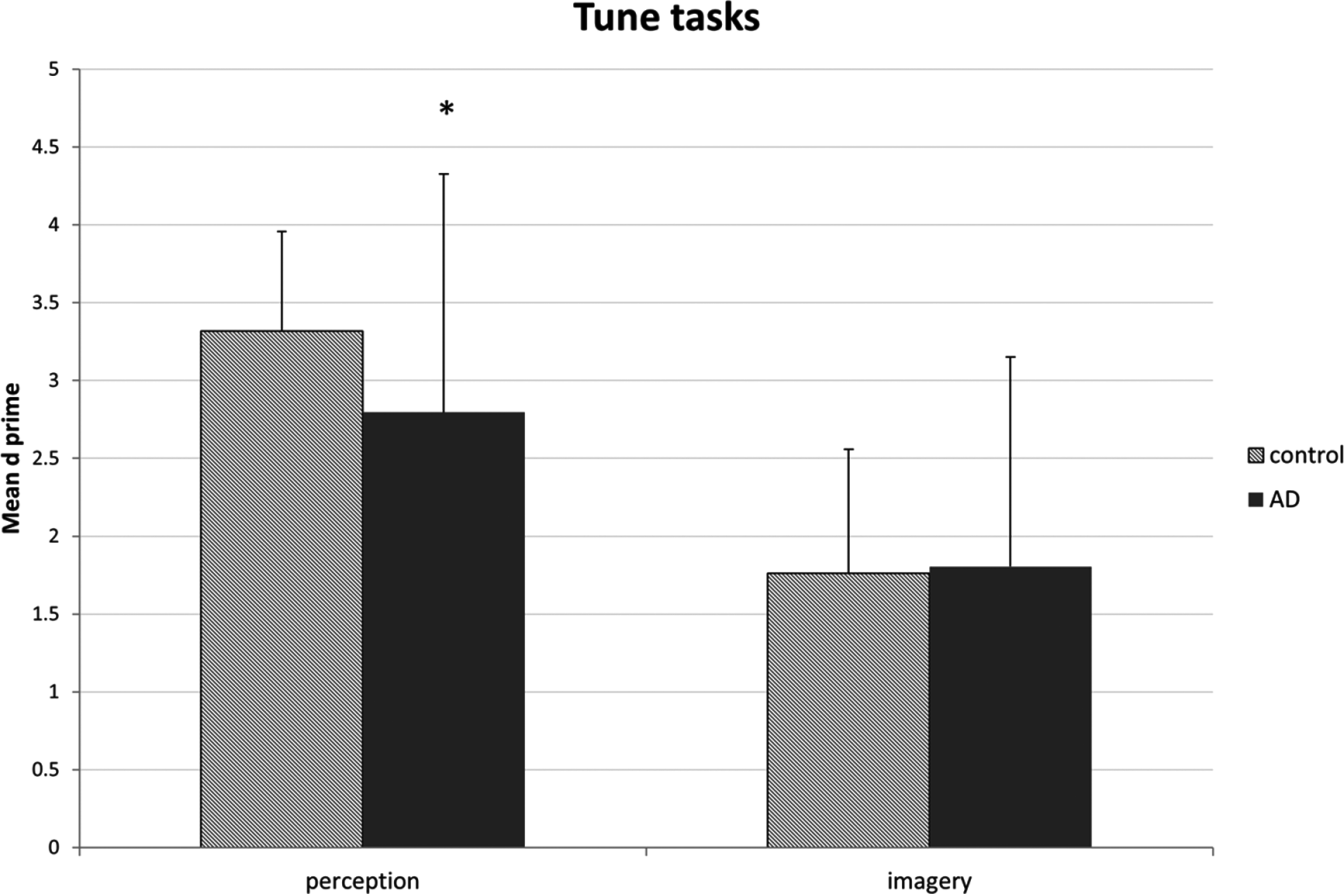
Accuracy in deciding if the second note is higher or lower than the first in perceived and imagined familiar songs, in Alzheimer's disease (AD) patients and controls. *P < 0.05.

The dependent measure was *d* ′. Patients did not differ significantly from controls in age (*t* = −1.43, *P* = 0.16), gender distribution (*χ*^2^ = 0.72, *P* = 0.40), or years of musical training (*t* = −1.7, *P* = 0.11). Years of musical training significantly affected *d* ′ scores (*β* = 0.20, CI = 0.1–0.3, *P* = 0.01), but age did not (*β* = 0.04, CI = −0.05 to 0.1, *P* = 0.39). Condition had a significant effect on *d* ′ scores (*β* = −1.56, CI = −2.2 to −0.9, *P* < 0.001), as did group (*β* = −1.15, CI = −2.3 to −0.02, *P* = 0.046). There was no interaction between condition and group (*F*(1,25) = 0.77, *P* = 0.39). However, as an exploratory analysis, we compared the groups on both tasks. The patients were clearly equivalent to the controls on imagery but were less accurate than the controls in the perceptual version (which was the easier task; Fig.3).

## Discussion

We begin by noting that data collection is not complete, so our results are tentative, based as they are on small samples of patients. As in all clinical research, accrual of patients is challenging because of the need to verify diagnoses using gold-standard criteria as well as getting cooperation from patients and families. In addition, our tests required that all participants have some basic musical abilities, but on the other hand, we did not want a sample of professional musicians (some of whom showed up in early recruitment), and we furthermore matched musical background between the groups.

These preliminary results for the Garner (musical dimensions) task showed that accuracies did not differ between the groups, which assures us that the PDD/DLB patients understood the task. Latency of response is of more interest. Normalized for baseline speed, patients were slower on pitch judgments and faster on timbre judgments in noninterference conditions. More germane to the hypothesis, the only reliable difference between the groups in an interference condition was in timbre judgments when pitch was changing (an example of impairment of suppression), in the mixed condition (which required set-shifting). Thus, early evidence suggests that PDD/DLB patients are impaired in disentangling timbre from pitch judgments when they also have to switch strategies. The timbre judgments were more difficult than pitch judgments for everyone (main effect of condition in accuracies), so that we see the deficit in the condition that presented the most challenges.

In the auditory imagery task, AD participants scored lower than controls overall. Although the group-by-condition interaction was not significant, we noted a trend in that mean scores on the perception version were lower in the patients. Should this trend become stronger with larger samples, that outcome would be consistent with prior literature,^22^ showing that patients have some auditory perception deficits. However, as yet we see no trend toward lower performance on the more difficult imagined pitch task (group performance was above chance levels). We can exclude the possibility that we had unusually mildly affected patients, as their scores on the usual neuropsychological tests including working memory were in the typical range for patients. Thus, if we continue to see group equivalence on the imagery task, we would conclude that the multimodal nature of imagined tasks (involving memory and perceptual circuits) may be offering sufficient neural support to compensate for deficits seen in more unimodal memory tasks.

We also want to caution that these two syndromes can co-occur, so ultimately we hope to conduct studies with larger samples from both syndromes and on both tasks. We would hypothesize double dissociations between the perceptual–memory task and the executive task if the dementias affect, as we think, two fundamentally different aspects of cognitive behavior.

Finally, we hope we have offered encouraging preliminary rationale for further systematic study of customized music cognition tests as probes of function in dementia syndromes. Musical tasks can supplement the traditional emphasis on verbal and visual cognition to help us understand the spectrum of these disorders. And because most people like music, we offer the prospect of using this information to help develop therapeutic interventions using enjoyable and engaging material.
